# Clinical Value of Routine Preoperative Ultrasonography in Bariatric Surgery Candidates: A Retrospective Analysis of 1119 Cases

**DOI:** 10.3390/tomography11110129

**Published:** 2025-11-14

**Authors:** Sangar Abdullah, Güney Özkaya, Adnan Gündoğdu, Murat Şendur

**Affiliations:** 1 Sancaktepe Prof. Dr. Ilhan Varank Teaching and Research Hospital, Istanbul 34885, Türkiye; guneyozkaya4@outlook.com (G.Ö.); adnangundogdu89@gmail.com (A.G.); 2 Ataşehir Central Hospital, Istanbul 34758, Türkiye; muratsendur@gmail.com

**Keywords:** bariatric surgery, ultrasonography, preoperative evaluation, surgical cancellation, obesity, hepatobiliary disease

## Abstract

**Background:** Preoperative evaluation in bariatric surgery aims to minimize perioperative risks and identify comorbid abdominal pathologies that may influence surgical planning. The role of routine abdominal ultrasonography (USG) remains debatable. **Methods:** This retrospective study included 1119 consecutive candidates for bariatric surgery who underwent routine preoperative ultrasonography (USG) between January 2022 and October 2024. Patients were stratified by BMI and categorized according to USG findings as normal, incidental, requiring follow-up/concomitant procedures, or necessitating cancellation. Baseline characteristics, USG findings, surgical outcomes, and predictors of cancellation were analyzed using univariate, multivariate, and Firth’s penalized logistic regression analyses. Ultrasonographic findings were further stratified as clinically significant (requiring intervention) or non-clinically significant (not requiring intervention) to standardize interpretation. **Results:** Abnormal USG findings were present in 77.5% of patients, with hepatic steatosis (60.8% [n = 680]), hepatomegaly (21.5%), and gallstones (13.9%) being the most frequent. Higher BMI was significantly associated with hepatomegaly, steatosis, and gallstones (all *p* < 0.05), but not with surgical cancellation. Bariatric surgery was cancelled in 11 patients (1.0%) due to critical findings exclusively identified on USG, including large ovarian/uterine masses, choledochal cysts, and suspected malignancies. In multivariate and Firth-adjusted regression, large ovarian/uterine masses (adjusted OR 12.9, 95% CI 3.0–55.2, *p* = 0.001; Firth OR 11.4, 95% CI 2.5–51.4, *p* = 0.002) and choledochal cysts (Firth OR 29.7, 95% CI 1.8–489.5, *p* = 0.048) emerged as independent predictors of cancellation. **Conclusions:** Although the overall cancellation rate was low, the detection of critical USG findings in 1.0% of patients had major clinical implications, preventing inappropriate or unsafe surgery and enabling timely referral for specialist management. Routine preoperative ultrasonography thus offers a clinically meaningful safeguard in bariatric surgery, supporting its inclusion in preoperative assessment algorithms.

## Simple Summary

People preparing for obesity surgery need careful health checks before the operation, but some doctors debate if a routine abdominal ultrasound is needed. In this study, we reviewed ultrasound results from 1119 patients before obesity surgery. Most results showed common findings such as gallbladder changes; however, about 1% of patients had critical problems, leading to the cancellation of their surgery or requiring follow-up. These results support the use of ultrasound as a simple and affordable test that can help identify important conditions and guide better surgical planning and patient care.

## 1. Introduction

Obesity, defined as a body mass index (BMI) of ≥30 kg/m^2^, has become a serious global health issue, with obese populations steadily increasing. According to the World Health Organization, worldwide obesity has more than tripled since 1975, with 890 million adults classified as obese in 2022 (World Health Organization 2025). Morbid obesity is associated with multiple comorbidities, including gastrointestinal and hepatobiliary disorders, hypertension, type 2 diabetes mellitus, cardiovascular diseases, various cancers, and obstructive sleep apnea [1]. Obesity accounts for an estimated 4–8 million annual deaths from obesity-associated morbidities. This epidemic also carries a substantial economic burden, with obesity-related healthcare costs exceeding $2 trillion annually worldwide [2].

Metabolic and bariatric surgery (MBS) is recommended for individuals with BMI of 35 kg/m^2^ or higher, regardless of the presence, absence, or severity of comorbidities. MBS should be considered for individuals with metabolic disease and a BMI of 30–34.9 kg/m^2^ [3]. Metabolic and bariatric surgery remains the most effective intervention for achieving durable weight loss and improving obesity-related comorbidities [4]. Procedures such as sleeve gastrectomy, Roux-en-Y gastric bypass, one-anastomosis gastric bypass, and newer procedures, including single-anastomosis sleeve ileal bypass (SASI), have been shown to induce weight loss and achieve remission of hypertension, type 2 diabetes mellitus, and other obesity-associated comorbidities [5]. Nevertheless, optimizing preoperative assessment is essential to reduce perioperative risk, guide surgical decision-making, and identify concurrent abdominal pathologies that may alter the surgical plan [4,6]. According to the International Federation for the Surgery of Obesity and Metabolic Disorders (IFSO) and the World Gastroenterology Organization (WGO) consensus guidelines on obesity management, preoperative assessment involves a multidisciplinary team. At a minimum, this team includes an obesity physician, a bariatric surgeon, a dietitian, and behavioral health professionals. It may also include subspecialty providers such as cardiologists, pulmonologists, endocrinologists, and anesthesiologists [1,7], a protocol we adhere to in our practice. After a comprehensive medical history and physical examination, a metabolic assessment is undertaken to identify and assess comorbidities [8]. The metabolic assessment includes complete blood count and serum levels of creatinine, liver enzymes, lipids, thyroid-stimulating hormone, and either hemoglobin A1C or fasting plasma glucose. Nutritional evaluations should include an iron panel, vitamin D, calcium, parathyroid hormone (PTH), vitamin B12, folic acid, and albumin. Patients undergoing malabsorptive surgeries should also have serum levels of vitamins A and E measured, while gastric bypass patients should be screened for Helicobacter pylori. Trace elements (zinc, copper, and selenium) can also be considered prior to malabsorptive surgeries. Comprehensive preoperative assessment also includes cardiac evaluation, given the prevalence of cardiovascular comorbidities in obese population. After initial clinical assessment and electrocardiography, echocardiography is recommended for patients with cardiovascular risk factors or symptoms to stratify perioperative risk [9,10]. At our institution, cardiology consultation and echocardiography are routinely per-formed for all bariatric surgery candidates.

Abdominal imaging studies play a crucial role in preoperative assessment, upper gastrointestinal series and endoscopy are commonly performed, and the role of abdominal ultrasonography (USG) remains contentious [1,8,11]. The IFSO and WGO Delphi survey on preparatory steps prior to metabolic and bariatric surgery (MBS) reached consensus on several key points. These include the need for comprehensive medical and nutritional evaluations, identifying and correcting all nutritional deficiencies, smoking cessation, and preoperative endoscopy, with sleep apnea screening required only for those at high risk. However, there was no consensus on routine preoperative imaging, and experts disagreed on the routine requirement for CT or MRI to screen for hepatocellular carcinoma prior to MBS [7].

Abdominal ultrasonography (USG) is a safe, non-invasive, cost-effective, and easily performed imaging method that enables the evaluation of the liver, pancreas, gallbladder, kidneys, and spleen. Gallstones, non-alcoholic fatty liver disease (NAFLD), hepatomegaly, and renal cysts are commonly associated with obesity. Rapid postoperative weight loss following bariatric surgery further predisposes patients to gallstone formation, a complication that may necessitate subsequent cholecystectomy [8,12]. Despite these benefits, the use of routine preoperative USG in asymptomatic bariatric candidates remains controversial with conflicting evidence. For example, recent studies report widely varying rates of clinically significant findings: Hany et al. [6]) found that while 70% had abnormal USG findings, only 2.3% required surgical plan modification, highlighting both the strengths and limitations of routine preoperative USG. In contrast, Abu Hussein et al. [13] suggested that preoperative USG should be selective and limited to only symptomatic patients. Almazeedi et al. [14] reported that USG does not add significant information to the preoperative workup and should be reserved for indicated patients. The American College of Radiology (ACR) appropriateness criteria further states that routine preoperative USG is usually not appropriate [15]. The IFSO and WGO consensus guidelines on obesity management recommend selective preoperative USG for patients with clinical suspicion of biliary disease or significantly elevated liver enzymes, but do not support routine imaging for all asymptomatic patients [7]. This distinction between selective and routine imaging highlights ongoing gaps in agreement and fuels significant variability in practice, particularly across regions where routine imaging is often deemed essential for safety [2]. However, in clinical practice, many experienced bariatric surgeons consider preoperative USG an essential component of preoperative assessment due to high prevalence of occult pathologies in this patient population.

Given the ongoing debate, the role of routine preoperative ultrasonography warrants further evaluation in well-defined patient cohorts. This study, therefore assessed the prevalence and clinical significance of abdominal ultrasonographic findings in a large series of bariatric surgery candidates, examining the spectrum of ultrasonographic abnormalities across BMI categories. We specifically investigated how USG results influenced surgical decision-making, including the need for concomitant procedures, postoperative follow-up, or cancellation of surgery. By addressing these questions, we aim to clarify the clinical value of preoperative ultrasonography in contemporary bariatric practice and provide evidence to guide future recommendations.

## 2. Materials and Methods

### 2.1. Study Design and Population

This retrospective observational study was conducted in two tertiary bariatric surgery centers and included a total of 1119 consecutive patients who underwent preoperative abdominal USG as part of the routine evaluation for bariatric surgery candidates. Patients were enrolled between January 2022 and October 2024. Demographic data, anthropometric measurements, surgical type, and detailed USG findings were extracted from institutional electronic records.

### 2.2. Grouping of Patients

Patients were stratified according to BMI into four categories: 35–40 kg/m^2^, 40–45 kg/m^2^, 45–50 kg/m^2^, and ≥50 kg/m^2^ (n = 77). In addition, based on USG results, patients were classified into four groups:

Group A (Normal): No abnormal USG findings;

Group B (Incidental): Findings not requiring further intervention;

Group C (Follow-up): Findings requiring additional evaluation or concomitant procedure;

Group D (Delay/Cancel): Findings leading to delay or cancellation of surgery.

For analytical purposes, Groups C and D were regarded as clinically significant, as they required either therapeutic modification or postponement of surgery, whereas Groups A and B were categorized as non-clinically significant, reflecting findings that did not necessitate intervention. This hierarchical grouping was designed to facilitate a uniform interpretation of ultrasonographic outcomes across study centers and to facilitate correlation analyses with surgical decision-making.

### 2.3. Ultrasonographic Evaluation

All preoperative USG examinations were performed within the same institutional radiology department by a dedicated team of board-certified radiologists, each with more than five years of experience in abdominal imaging. To ensure consistency, all examinations were interpreted and reported by this radiology team using predefined institutional diagnostic criteria.

The imaging protocol was standardized across both study sites. USG scans were performed using high-resolution abdominal probes (3.5–5 MHz convex transducers, GE Logic E9, GE Healthcare, Chicago, IL, USA). Patients were examined in the supine and left lateral decubitus positions after a minimum fasting period of 8 h to optimize gallbladder and hepatobiliary evaluation.

The examination protocol routinely included evaluation of the liver, gallbladder, spleen, pancreas, kidneys, urinary bladder, adrenal glands, and, in female patients, the uterus and ovaries. The interpretation of ultrasonographic findings was performed not only by anatomical localization but also by their potential impact on surgical management. In line with current diagnostic conventions, conditions identified during preoperative abdominal ultrasonography were conceptually divided into two categories: those of clinical significance, defined as findings necessitating therapeutic intervention, additional diagnostic evaluation, or modification of the surgical plan; and those of non-clinical significance, which did not require intervention but were recorded for completeness or longitudinal follow-up. This dual-tiered framework was adopted to standardize radiologic reporting between centers and to ensure alignment between imaging outcomes and surgical decision-making. Hernias and other rare pathologies were also recorded. Abnormalities were classified according to their clinical relevance as incidental findings, as requiring follow-up or concomitant procedures, or leading to cancellation or delay.

### 2.4. Surgical Procedures

All bariatric operations were performed by experienced bariatric surgeons within the same surgical team, ensuring standardized perioperative management and technical uniformity. The predominant procedure was laparoscopic sleeve gastrectomy, performed as the primary bariatric procedure. In selected cases where concomitant gallbladder pathology was identified during preoperative assessment, sleeve gastrectomy was combined with laparoscopic cholecystectomy, allowing simultaneous treatment of biliary disease without the need for an additional operation.

A smaller subset of patients underwent single-anastomosis sleeve ileal bypass (SASI), offered according to individual patient characteristics and surgical indications. All procedures were performed using standard laparoscopic techniques, with careful attention to intraoperative safety and enhanced recovery protocols.

### 2.5. Statistical Analysis

Continuous variables were tested for normality using the Shapiro–Wilk test and expressed as median and interquartile range (IQR). Categorical variables were presented as absolute numbers and percentages. Comparisons across BMI categories and USG groups were performed using the Kruskal–Wallis test for continuous variables and the Pearson chi-square test or Fisher’s exact test for categorical variables, as appropriate. A *p*-value < 0.05 was considered statistically significant. Statistical analyses were conducted using SPSS version 22 (IBM Corp., Armonk, NY, USA).

## 3. Results

### 3.1. Baseline Characteristics of the Study Cohort

Baseline demographic and surgical characteristics were evaluated according to BMI categories. The median age of the entire cohort was 38 years (IQR 31–46) and did not differ significantly across BMI categories (*p* = 0.18). Male patients constituted 18.5% (n = 207) of the cohort, with no significant variation among BMI subgroups (*p* = 0.64). When stratified by USG-based groups, age differed significantly, being higher in Group C (41 years, IQR 32–49) compared to Group A (35 years, IQR 28–42) (*p* < 0.001). Sex distribution also varied significantly among groups, with the proportion of males ranging from 11.5% in Group A to 22.2% in Group C (*p* = 0.005). Median BMI was 41.5 kg/m^2^ (IQR 39.0–45.0) overall, and significantly higher in Group D (44.0, [IQR 41.3–45.9]) compared with other groups (*p* < 0.001). The distribution of USG groups across BMI categories showed significant differences (*p* < 0.001). The proportion of patients with normal USG findings (Group A) decreased progressively with increasing BMI, from 29.4% in BMI 35–40 kg/m^2^ to 10.4% in BMI ≥ 50 kg/m^2^. Conversely, patients requiring follow-up or concomitant procedures (Group C) increased from 16.3% to 39.0% across the same BMI categories. The delay/cancellation group (Group D) was more frequent in the BMI ≥ 50 group (6.5%) compared with lower BMI strata. The type of surgery also differed significantly between BMI and USG groups (both *p* < 0.001). Overall, 97.4% (n = 1090) underwent sleeve gastrectomy and 1.6% (n = 18) underwent SASI, while 1.0% (n = 11) were cancelled preoperatively. Cancellation was observed exclusively in Group D, with 100% of these patients not proceeding to surgery. Other baseline variables did not show significant differences. These findings are presented in Table 1.

### 3.2. Ultrasonographic Findings by BMI Categories

Ultrasonographic findings were systematically evaluated across BMI subgroups. Hepatomegaly was detected in 240 patients (21.5%), with prevalence increasing from 15.2% in BMI 35–40 kg/m^2^ to 32.5% in BMI ≥ 50 kg/m^2^ *p* < 0.001). Hepatic steatosis was the most frequent abnormality (680 patients, 60.8%) and was significantly associated with a higher BMI (56.4% in BMI 35–40 vs. 62.3% in BMI ≥ 50, *p* = 0.025). Gallstones were identified in 155 patients (13.9%), with prevalence rising to 24.7% in the BMI ≥50 subgroups (*p* = 0.002). Less common but statistically significant or borderline findings included splenomegaly in 17 patients (1.5%), bladder wall thickening (n = 1, 0.1%), diastasis recti (n = 2, 0.2%), and suspected lymphoma (n = 2, 0.2%) (all *p* < 0.05). Other USG abnormalities, including gallbladder polyps, liver hemangiomas, focal fatty sparing, renal stones, renal cysts, uterine myomas, ovarian cysts, and various hernias, were observed but did not differ significantly among BMI subgroups. Detailed results are presented in Table 2.

### 3.3. Ultrasonographic Findings by USG Groups

USG findings were also stratified according to clinical groups (B: incidental, C: follow-up/concomitant, D: delay/cancellation). When stratified by USG-based clinical groups, significant differences were observed for several ultrasonographic findings. Hepatomegaly was identified in 240 patients (27.7%) and was more frequent in Group C (30.4%) and Group D (43.5%) compared to with Group B (25.6%) (*p* < 0.001). Hepatic steatosis was detected in 680 patients (78.4%), and showed a higher prevalence in Group D (87.0%) compared with Groups B (79.9%) and C (73.9%) (*p* < 0.001). Gallstones were identified in 155 patients (17.9%) and were significantly more frequent in Groups C (17.4%) and D (21.7%) than in Group B (17.6%) (*p* = 0.002). Rare but clinically critical findings, such as choledochal cyst (n = 1, *p* = 0.041), adrenal lesions > 4 cm (n = 1, *p* = 0.074), pancreatic lesions (n = 1), ovarian solid lesions (n = 1), and suspected lymphoma (n = 2, *p* = 0.051) were confined to Group D patients. Uterine fibroids (n = 16, 1.8%), ovarian cysts (n = 14, 1.6%), and umbilical hernias (n = 14, 1.6%) were also more common in Group C and D patients compared with Group B, with umbilical hernia reaching statistical significance (*p* = 0.014). These data are summarized in Table 3.

### 3.4. Predictors of Surgery Cancellation

Regression analyses were performed to identify independent predictors of bariatric surgery cancellation (Group D). In univariate analysis, multiple USG findings showed significant association with cancellation: large ovarian/uterine masses (OR 17.0, 95% CI 5.3–54.5, *p* < 0.001), hepatomegaly (OR 38.2, 95% CI 18.9–77.1, *p* < 0.001), hepatic steatosis (OR 6.3, 95% CI 1.3–30.0, *p* = 0.008), gallstones (OR 5.3, 95% CI 1.8–12.9, *p* = 0.002), and choledochal cyst (OR 29.7, 95% CI 2.3–Not estimable, *p* = 0.041), while suspected lymphoma (*p* = 0.051) showed borderline significance. large ovarian/uterine masses remained significant (adjusted OR 12.9, 95% CI 3.0–55.2, *p* = 0.001), while bladder polypoid lesion (OR 12.4, 95% CI 0.7–220.1, *p* = 0.093) and adrenal lesion > 4 cm (OR 15.8, 95% CI 0.8–312.5, *p* = 0.074) showed borderline associations. Firth’s penalized logistic regression confirmed large ovarian/uterine masses (OR 11.4, 95% CI 2.5–51.4, *p* = 0.002) and choledochal cyst (OR 29.7, 95% CI 1.8–489.5, *p* = 0.048) as independent predictors of cancellation, while liver mass/metastasis (OR 35.8, 95% CI 0.8–1600, *p* = 0.073), bladder polypoid lesion (OR 10.9, 95% CI 0.6–192.5, *p* = 0.110), and adrenal lesion > 4 cm (OR 13.6, 95% CI 0.7–271.4, *p* = 0.089) did not reach statistical significance. These analyses are presented in Table 4.

## 4. Discussion

Routine preoperative USG yielded a low overall cancellation rate in our bariatric cohort, but the clinical significance of those cancellations is high. All 11 patients in the cancellation group harbored critical, occult pathologies—such as choledochal cysts, large ovarian/uterine masses, or suspicious tumors—that were only detected on ultrasound. These findings would not have been predicted by BMI or standard clinical screening, yet they necessitated deferral of bariatric surgery for definitive management. Identifying these conditions preoperatively enabled timely specialist referral and avoided proceeding with bariatric surgery under potentially perilous circumstances. In essence, although uncommon, detecting Group D abnormalities beforehand represents a clinically meaningful prevention of serious perioperative complications in those 1% of patients. Our results underscore that even a 1.0% cancellation rate can translate into substantial patient safety benefits, given the severity of issues averted.

When evaluating our results, it is crucial to consider global variability in preoperative imaging methods for MBS. The 2005 European Association for Endoscopic Surgery (EAES) guidelines explicitly included abdominal ultrasonography as part of the standard preoperative evaluation [16]; however, the 2020 EAES update emphasizes customized preoperative evaluation rather than explicitly requiring routine USG [17]. Most notably, the recently updated IFSO and WGO guidelines on obesity management recommend selective preoperative USG only for patients with clinical suspicion of biliary disease or significantly elevated liver enzymes, and do not support routine imaging for asymptomatic patients [7]. A recent study by Lesourd et al. [18] concluded that routine abdominal CT scans were not necessary for preoperative evaluation, although they may play a selective role in patients with significant hiatal hernias. Despite these international recommendations favoring selective imaging, clinical practice varies considerably across regions. In many countries, particularly in Europe, the Middle East, and Asia, routine preoperative imaging, particularly USG, remains widely implemented as a standard clinical practice. This disconnect between international guideline recommendations and regional practice often reflects medico-legal considerations and defensive medicine practices, where routine imaging has become a practical necessity regardless of evidence-based guidelines. A survey answered by 93 surgeon members of the Pan Arab Society of Metabolic and Bariatric Surgery (PASMBS) revealed that 63% routinely performed USG, but no formal consensus exists [19]. Studies from Asia and the Middle East have documented widespread use of comprehensive preoperative assessment, including endoscopy for screening [20,21]. In the absence of national Turkish guidelines on preoperative imaging for MBS, our approach in Turkey aligns with this regional approach, implementing routine preoperative USG while reserving CT for selective indications.

In our practice, CT is considered essential in specific circumstances where USG is insufficient or technically limited. CT is reserved for the following: 1—USG that is technically inadequate and yields inconclusive results due to extreme obesity and excessive bowel gas limiting proper view; 2—USG identifies or raises suspicion for complex intra-abdominal pathologies such as masses, anatomical variants, and vascular anomalies requiring further evaluation before surgery; 3—preoperative endoscopy identifies significant hiatal hernia requiring CT evaluation to determine the need for concomitant hernia repair [18]. While CT provides superior anatomical detail and overcomes the limitations of body habitus that can impair USG image quality [22,23,24], routine preoperative CT is not justified given the lack of demonstrated benefit in asymptomatic patients [18], radiation exposure concerns, and the higher cost compared to USG. Therefore, CT serves as a complementary tool reserved for selected cases where USG findings are indeterminate, or when anatomical questions cannot be adequately addressed by USG, rather than as a routine first-line imaging study.

These findings contribute to the ongoing debate on the utility of routine imaging before bariatric surgery [11,25,26]. Prior studies have reported that while preoperative ultrasound frequently uncovers abnormalities, it seldom changes the bariatric surgical plan [14]. For instance, Abou Hussein et al. noted abnormal ultrasound findings in 62.3% of 937 patients yet concluded that routine USG “does not seem to have an important part” in the preoperative workup [13]. Similarly, Almazeedi and colleagues found that 67.9% of 747 sleeve gastrectomy patients had some sonographic abnormality, as 57.2% hepatic steatosis and 11.1% cholelithiasis, but they observed no significant benefit to universal screening and advised reserving ultrasound for indicated cases [14]. These earlier reports emphasize the low yield of actionable findings and have led some to question the cost-effectiveness of routine imaging in asymptomatic bariatric candidates [6]. Indeed, routine ultrasound is time-consuming and can be technically challenging in morbidly obese individuals, and guidelines often limit its use to patients with symptoms or abnormal liver enzymes [14]. A focused, risk-factor guided approach has been suggested to optimize resource utilization [6,13,27].

Notwithstanding the above, our data—in line with more recent large-scale studies—illustrate the value of routine USG in detecting rare but consequential pathologies. In a cohort of 4418 bariatric patients who underwent preoperative ultrasound, Hany et al. reported significant findings in only 1.5% of cases; yet, roughly 0.3% of the total had their surgeries cancelled due to serious diagnoses that were incidentally discovered [6]. The study report noted that 15.9% of patients benefited from ultrasound, mainly through the detection of gallbladder disease, and an additional 1.4% had their bariatric procedure postponed specifically because critical abnormalities were found [6]. Similarly, Altıntaş and Bayrak reported that 2.1% of bariatric surgery candidates needed postponement or cancellation of their procedures due to preoperative USG results, which revealed doubtful liver lesions and other liver pathology necessitating additional study prior to surgery [28]. Nagarajan et al. also reported that ancillary findings on preoperative USG, including an obstructing ureteric stone, required stone removal prior to proceeding with surgery in 2.5% of their cohort [11]. While the majority of the patients in their study had common findings such as hepatosteatosis (51.8%) or cholelithiasis that did not alter the surgical plans, the detection of critical pathology in a small but significant number of patients aligns with our findings and reinforces the value of preoperative USG in identifying high-risk candidates who would otherwise proceed to surgery. Although infrequent, these anomalies—much like our Group D cases—represent “occult” conditions of high clinical significance. However, BMI was not a reliable predictor of surgery cancellation in our study, echoing previous observations that BMI correlates poorly with ultrasound findings, such as gallstones or fatty liver [2,11]. While these hepatobiliary findings are common, they did not independently predict cancellation. In practical terms, this means a purely selective imaging strategy based on BMI or routine labs could fail to detect the very patients at risk of preventable perioperative adverse events.

The present analysis also highlights how routine USG can enhance surgical planning for those with less critical findings. In this study, ultrasonographic findings were stratified according to clinical significance to facilitate uniform interpretation across centers. Groups C and D were designated as clinically significant, representing findings requiring therapeutic intervention, modification, or surgery postponement, whereas Groups A and B were classified as non-clinically significant, encompassing incidental findings without management implications. This stratification enabled a consistent framework for comparing radiologic findings with surgical decision-making and enhanced the interpretability of outcome analyses. Many patients had incidental conditions identified that, while not canceling surgery, prompted adjustments—for example, scheduling concomitant cholecystectomy for gallstones or arranging follow-up for benign liver lesions. In our cohort, higher BMIs were associated with a greater prevalence of hepatic steatosis, hepatomegaly, and cholelithiasis, which are expected sequelae of obesity. These common ultrasound findings did not alter the bariatric procedure itself, consistent with the notion that asymptomatic gallstones or fatty liver disease rarely contraindicate surgery [14,29]. Nonetheless, the ability to anticipate such issues is useful. Detecting gallstones preoperatively enables surgeons and patients to deliberate on whether to perform a concomitant cholecystectomy—a strategy supported by prior studies showing that up to 16–18% of bariatric surgery candidates have chronic cholecystitis or significant gallbladder disease detected on routine ultrasound or intraoperative assessment [6,30]. In our practice, ultrasound findings categorized as “follow-up or concomitant procedure” (Group C) facilitated a tailored approach, potentially reducing postoperative surprises and the need for interventions.

From a broader perspective, even a small percentage of cancellations or case modifications can have a significant impact in a high-volume bariatric program. Preventing 1–2 in every 100 patients from undergoing an ill-advised surgery is a patient safety win that may justify the routine use of a relatively low-cost, non-invasive test, such as ultrasonography. Our utilization of Firth’s penalized logistic regression—a method well-suited for rare-event analysis [31]—further confirmed that the presence of a critical ultrasound-detected finding was an independent predictor of surgery cancellation, with markedly increased odds, whereas patient BMI and other baseline factors showed no such association. This statistical confirmation lends weight to the argument that routine USG adds a unique layer of protection that standard evaluations alone do not provide.

Overall, the clinical significance of detecting Group D findings prior to bariatric surgery is substantial despite their relatively low incidence. Incorporating routine abdominal USG into the preoperative evaluation allows for the identification of otherwise occult but clinically critical conditions, thereby preventing unsafe surgical procedures. Our data suggest that approximately 1% of patients may avoid undergoing surgery under high-risk circumstances, such as in the presence of unrecognized malignancies or complex cystic lesions, solely because of this screening step. From a cost-effectiveness perspective, routine preoperative ultrasonography (USG) adds upfront costs; however, these are likely outweighed by expenses of managing missed critical findings, such as emergency interventions, prolonged hospitalizations, reoperations, or legal complications. Preoperative detection of gallbladder pathology also allows planned concomitant cholecystectomy, by avoiding future procedures for symptomatic cholelithiasis and offering both clinical and economic benefits.

Our regression analyses revealed that, in contrast to clinical or demographic factors like BMI, age, or sex, only certain USG-detected pathologies, particularly large ovarian or uterine masses and choledochal cysts, were predictors of surgery cancellation. Common findings, including hepatic steatosis, hepatomegaly, and gallstones, were not independent predictors of surgery cancellation, highlighting that serious, surgery-altering conditions are often clinically silent. In resource-limited settings, a selective imaging strategy targeting higher-risk patients may appear practical. Such an approach might focus on those with elevated liver enzymes, known hepatobiliary pathology, gynecological symptoms, or a history of abdominal surgery. However, it is important to note that these criteria were not derived from our data, and some patients with critical findings may be missed. Centers might adopt routine preoperative ultrasound given its safety net value, while continuing to refine criteria to identify those most likely to benefit. Prospective studies incorporating formal cost-effective analyses and clinical prediction models are needed to provide robust evidence and guide future practice. In conclusion, routine preoperative USG, despite frequently identifying incidental benign findings, provides an important opportunity to prevent potentially serious perioperative complications, contributing meaningfully to the safe conduct of bariatric surgery.

### Study Straightness and Limitations

This study has several strengths. It represents one of the larger two-center series evaluating routine preoperative ultrasonography in bariatric surgery, including over 1000 patients within a defined timeframe. The standardized imaging protocol, performed and interpreted by the same institutional radiology team, minimized inter-observer variability and enhanced diagnostic consistency. The application of advanced statistical methods, including Firth’s penalized logistic regression, ensured reliable estimation of predictors despite a few cancellation events. Nevertheless, some limitations exist. The retrospective design introduces potential biases in data completeness and patient selection. Although the sample size was large, the limited number of cancellation cases (n = 11) constrained the statistical power of subgroup analyses and restricted the variables in multivariate modeling.

## 5. Conclusions

In this large cohort of bariatric surgery candidates, routine preoperative ultrasonography revealed a wide spectrum of hepatobiliary, gynecologic, and abdominal wall abnormalities in 78.1% (n = 867). Although the overall cancellation rate was low (1.0%), all cancelled cases were attributable to critical, otherwise unsuspected findings such as choledochal cysts, large ovarian or uterine masses, or suspected malignancies. Early identification of these conditions prevented patients from undergoing inappropriate or high-risk surgery and enabled timely referral for definitive management.

While BMI correlated with common abnormalities, including hepatic steatosis, hepatomegaly, and gallstones, it was not an independent predictor of surgical cancellation. Instead, cancellation was determined exclusively by distinct ultrasound-detected pathologies.

These results highlight the clinical importance of incorporating routine ultrasonography into the preoperative assessment algorithm for bariatric patients. Even with a modest cancellation rate, the detection and prevention of potentially serious complications justify its use as a low-cost, non-invasive, and safety-enhancing tool in bariatric practice.

## Figures and Tables

**Table 1:** The baseline demographic and clinical characteristics of the study cohort stratified by BMI categories and USG finding groups.

**Variable**	**Overall (N = 1119)**	**BMI 35–40** **(N = 374)**	**BMI 40–45** **(N = 468)**	**BMI 45–50** **(N = 200)**	**BMI ≥ 50** **(N = 77)**	***p*-Value**
Age (years)	38 (31–46)	37 (30–45)	38 (31–47)	40 (32–48)	36 (30–44)	0.18
Sex						0.64
○ Male	207 (18.5%)	43 (11.5%)	95 (20.3%)	45 (22.5%)	24 (31.2%)	
○ Female	912 (81.5%)	331 (88.5%)	373 (79.7%)	155 (77.5%)	53 (68.8%)	
Group						<0.001
○ A	252 (22.5)	110 (29.4)	103 (22.0)	31 (15.5)	8 (10.4)	
○ B	626 (55.9)	203 (54.3)	283 (60.5)	105 (52.5)	35 (45.5)	
○ C	230 (20.6)	61 (16.3)	80 (17.1)	59 (29.5)	30 (39.0)	
○ D	11 (1.0)	0 (0.0)	1 (0.2)	5 (2.5)	5 (6.5)	
Surgery type						<0.001
○ Sleeve	1090 (97.4%)	374 (100%)	458 (97.9%)	195 (97.5%)	63 (81.8%)	
○ SASI	18 (1.6%)	0 (0.0%)	10 (2.1%)	5 (2.5%)	3 (3.9%)	
○ Cancelled	11 (1.2%)	0 (0.0%)	0 (0.0%)	0 (0.0%)	11 (14.3%)	
**Variable**	**Overall (N = 1119)**	**Group A (Normal, N = 252)**	**Group B (Incidental, N = 626)**	**Group C (Follow-up, N = 230)**	**Group D (Delay/Cancel, N = 11)**	***p*-value**
Age (years)	38 (31–46)	35 (28–42)	38 (31–46)	41 (32–49)	35 (30–43)	<0.001
Sex						0.005
○ Male	207 (18.5%)	29 (11.5%)	125 (20.3%)	50 (21.7%)	1 (9.1%)	
○ Female	912 (81.5%)	223 (88.5%)	499 (79.7%)	180 (78.3%)	10 (90.9%)	
BMI (kg/m^2^)	41.5 (39.0–45.0)	40.0 (37.7–42.2)	42.1 (39.6–45.8)	42.0 (39.0–45.0)	44.0 (41.3–45.9)	<0.001
Surgery type						<0.001
○ Sleeve	1088 (97.2%)	250 (99.2%)	616 (98.4%)	222 (96.5%)	0 (0.0%)	
○ SASI	18 (1.6%)	2 (0.8%)	10 (1.6%)	6 (2.6%)	0 (0.0%)	
○ Cancelled	11 (1.2%)	0 (0.0%)	0 (0.0%)	0 (0.0%)	11 (100%)	

Data are presented as median (Q1–Q3) for continuous variables and n (%) for categorical variables. *p*-values calculated using the Kruskal–Wallis test for continuous variables and Pearson’s chi-square or Fisher’s exact test for categorical variables. Group A = Normal USG; Group B = Incidental findings (no surgical change), non-clinically significant; Group C = Findings requiring follow-up or concomitant procedure, clinically significant; Group D = Findings requiring delay or cancellation of surgery, clinically significant. BMI, body mass index; SASI, single-anastomosis sleeve ileal bypass.

**Table 2:** Distribution of Preoperative Abdominal Ultrasonography Findings According to BMI subgroups.

Findings (USG)	Overall (N = 1119)	BMI 35–40 (N = 374)	BMI 40–45 (N = 468)	BMI 45–50 (N = 200)	BMI ≥ 50 (N = 77)	*p*-Value
Normal USG	252 (23.5%)	104 (29.4%)	98 (22.0%)	31 (15.5%)	19 (24.7%)	<0.001
**Hepatobiliary**						
Hepatomegaly	240 (21.5%)	57 (15.2%)	98 (21.0%)	60 (30.0%)	25 (32.5%)	<0.001
Hepatic steatosis	680 (61.7%)	211 (56.4%)	307 (65.6%)	124 (62.0%)	48 (62.3%)	0.025
Gallstone	155 (13.9%)	37 (9.9%)	67 (14.3%)	32 (16.0%)	19 (24.7%)	0.002
Gallbladder polyp	15 (1.3%)	5 (1.3%)	6 (1.3%)	2 (1.0%)	2 (2.6%)	0.809
Liver hemangioma	20 (1.8%)	2 (0.8%)	7 (1.1%)	9 (3.9%)	2 (18.2%)	0.421
Simple liver cyst	10 (0.9%)	2 (0.5%)	5 (1.1%)	2 (1.0%)	1 (1.3%)	0.882
Liver hydatid cyst	2 (0.2%)	0 (0.0%)	0 (0.0%)	2 (1.0%)	0 (0.0%)	0.019
Liver metastatic lesion	1 (0.1%)	0 (0.0%)	0 (0.0%)	1 (0.5%)	0 (0.0%)	0.202
Focal fatty sparing lesion	5 (0.4%)	1 (0.3%)	2 (0.4%)	1 (0.5%)	1 (1.3%)	0.603
Choledochal cyst	1 (0.1%)	0 (0.0%)	0 (0.0%)	0 (0.0%)	1 (1.3%)	0.052
Accessory spleen	5 (0.4%)	3 (0.8%)	1 (0.2%)	1 (0.5%)	0 (0.0%)	0.347
Splenomegaly	17 (1.5%)	6 (1.6%)	5 (1.1%)	4 (2.0%)	2 (2.6%)	0.692
Pancreatic lesion	1 (0.1%)	1 (0.3%)	0 (0.0%)	0 (0.0%)	0 (0.0%)	0.364
**Urinary**						
Renal stone	32 (2.9%)	8 (2.1%)	13 (2.8%)	8 (4.0%)	3 (3.9%)	0.559
Simple renal cyst	27 (2.4%)	8 (2.1%)	8 (1.7%)	7 (3.5%)	4 (5.2%)	0.228
Renal angiomyolipoma	2 (0.2%)	1 (0.3%)	1 (0.2%)	0 (0.0%)	0 (0.0%)	0.778
Urinary bladder polypoid lesion	1 (0.1%)	0 (0.0%)	0 (0.0%)	1 (0.5%)	0 (0.0%)	0.202
Bladder wall thickness	1 (0.1%)	0 (0.0%)	0 (0.0%)	0 (0.0%)	1 (1.3%)	0.049
BPH	5 (0.4%)	0 (0.0%)	2 (0.4%)	2 (1.0%)	1 (1.3%)	0.079
Hypertrophic column of Bertin	1 (0.1%)	1 (0.3%)	0 (0.0%)	0 (0.0%)	0 (0.0%)	0.364
Grade I nephropathy	1 (0.1%)	0 (0.0%)	0 (0.0%)	1 (0.5%)	0 (0.0%)	0.202
Adrenal lesion (>4 cm)	1 (0.1%)	0 (0.0%)	1 (0.2%)	0 (0.0%)	0 (0.0%)	0.419
**Gynecologic**						
Uterine myoma	26 (2.3%)	8 (2.1%)	14 (3.0%)	3 (1.5%)	1 (1.3%)	0.319
Ovarian cyst	20 (1.8%)	5 (1.3%)	7 (1.5%)	6 (3.0%)	2 (2.6%)	0.218
Polycystic ovary	5 (0.4%)	2 (0.5%)	2 (0.4%)	1 (0.5%)	0 (0.0%)	0.873
Ovarian solid lesion	1 (0.1%)	0 (0.0%)	1 (0.2%)	0 (0.0%)	0 (0.0%)	0.419
**Hernia**						
Umbilical hernia	14 (1.3%)	3 (0.8%)	5 (1.1%)	4 (2.0%)	2 (2.6%)	0.301
Inguinal hernia	2 (0.2%)	0 (0.0%)	1 (0.2%)	1 (0.5%)	0 (0.0%)	0.580
Incisional hernia	2 (0.2%)	1 (0.3%)	1 (0.2%)	0 (0.0%)	0 (0.0%)	0.778
Femoral hernia	1 (0.1%)	0 (0.0%)	1 (0.2%)	0 (0.0%)	0 (0.0%)	0.419
Diastasis recti	2 (0.2%)	0 (0.0%)	0 (0.0%)	1 (0.5%)	1 (1.3%)	0.042
**Other**						
Lymphoma (suspected)	2 (0.1%)	0 (0.0%)	0 (0.0%)	0 (0.0%)	2 (1.3%)	0.052

Data are presented as n (%) for categorical variables. Between-group comparisons were performed using the Kruskal–Wallis test for continuous variables and Pearson’s chi-square or Fisher’s exact test for categorical variables, as appropriate. A *p*-value < 0.05 was considered statistically significant. BMI, body mass index; USG, ultrasonography; SASI, single-anastomosis sleeve ileal bypass; BPH, benign prostatic hyperplasia.

**Table 3:** Distribution of Preoperative Abdominal Ultrasonography Findings Stratified by USG-Based Clinical Groups.

USG Finding	Overall (N = 867)	Group B (N = 626)	Group C (N = 230)	Group D (N = 11)	*p*-Value
**Hepatobiliary system**					
Hepatomegaly	240 (27.7%)	160 (25.6%)	70 (30.4%)	10 (43.5%)	<0.001
Hepatic steatosis	680 (78.4%)	500 (79.9%)	170 (73.9%)	10 (87.0%)	<0.001
Gallstone	155 (17.9%)	110 (17.6%)	40 (17.4%)	5 (21.7%)	0.002
Gallbladder polyp	15 (1.7%)	10 (1.6%)	4 (1.7%)	1 (4.3%)	0.393
Liver hemangioma	20 (2.3%)	13 (2.1%)	6 (2.6%)	1 (4.3%)	0.934
Hydatid cyst	2 (0.2%)	1 (0.2%)	1 (0.4%)	0 (0.0%)	0.419
Simple liver cyst	10 (1.2%)	7 (1.1%)	2 (0.9%)	1 (4.3%)	0.882
Liver Metastatic lesion	1 (0.1%)	0 (0.0%)	0 (0.0%)	1 (4.3%)	0.202
Focal fatty sparing	5 (0.6%)	3 (0.5%)	2 (0.9%)	0 (0.0%)	0.419
Choledochal cyst	1 (0.1%)	0 (0.0%)	0 (0.0%)	1 (4.3%)	0.041
Splenomegaly	17 (2.0%)	11 (1.8%)	5 (2.2%)	1 (4.3%)	0.692
Accessory spleen	5 (0.6%)	3 (0.5%)	1 (0.4%)	1 (4.3%)	0.347
Pancreatic lesion	1 (0.1%)	0 (0.0%)	0 (0.0%)	1 (4.3%)	0.243
**Urinary system**					
Renal stone	32 (3.7%)	22 (3.5%)	8 (3.5%)	2 (8.7%)	0.559
Renal cyst	27 (3.1%)	18 (2.9%)	8 (3.5%)	0 (0.0%)	0.228
Renal angiomyolipoma	2 (0.2%)	1 (0.2%)	0 (0.0%)	0 (0.0%)	0.342
Bladder polypoid lesion	1 (0.1%)	0 (0.0%)	0 (0.0%)	1 (4.3%)	0.419
Bladder wall thickening	1 (0.1%)	0 (0.0%)	1 (0.4%)	1 (4.3%)	0.253
BPH	5 (0.6%)	3 (0.5%)	1 (0.4%)	1 (4.3%)	0.052
Hypertrophic column of Bertin	1 (0.1%)	1 (0.2%)	0 (0.0%)	0 (0.0%)	0.365
Grade I nephropathy	1 (0.1%)	0 (0.0%)	1 (0.4%)	0 (0.0%)	0.423
Adrenal lesion (>4 cm)	1 (0.1%)	0 (0.0%)	0 (0.0%)	1 (4.3%)	0.243
**Gynecologic system**					
Uterine myoma	16 (1.8%)	9 (1.4%)	4 (1.7%)	3 (27.3%)	0.319
Ovarian cyst (>5 cm)	14 (1.6%)	9 (1.4%)	5 (2.2%)	1 (9.1%)	0.218
Polycystic ovary	3 (0.3%)	2 (0.3%)	1 (0.4%)	0 (0.0%)	0.202
Ovarian solid lesion	1 (0.1%)	0 (0.0%)	0 (0.0%)	1 (9.1%)	0.265
**Hernias**					
Umbilical hernia	14 (1.6%)	9 (1.4%)	4 (1.7%)	1 (4.3%)	0.014
Inguinal hernia	2 (0.2%)	1 (0.2%)	1 (0.4%)	0 (0.0%)	0.623
Incisional hernia	2 (0.2%)	1 (0.2%)	1 (0.4%)	0 (0.0%)	0.419
Femoral hernia	1 (0.1%)	1 (0.2%)	0 (0.0%)	0 (0.0%)	0.419
Diastasis recti	2 (0.2%)	1 (0.2%)	1 (0.4%)	0 (0.0%)	0.041
**Other**					
Lymphoma (suspected)	2 (0.2%)	1 (0.2%)	0 (0.0%)	1 (4.3%)	0.051

Data are presented as n (%) for categorical variables. Between-group comparisons were performed using the Pearson chi-square test or Fisher’s exact test for categorical variables, as appropriate. A *p*-value <0.05 was considered statistically significant. BMI, body mass index; USG, ultrasonography; SASI, single-anastomosis sleeve ileal bypass; BPH, benign prostatic hyperplasia.

**Table 4:** Logistic regression analysis of ultrasonographic findings associated with bariatric surgery cancellation (Group D, n = 11).

Variable (Predictors)	Univariate OR (95% CI)	*p*-Value	Multivariate OR (95% CI)	*p*-Value	Firth Adjusted OR (95% CI)	*p*-Value
Hepatomegaly	38.2 (18.9–77.1)	<0.001	–	–	–	–
Hepatic steatosis	6.3 (1.3–30.0)	0.008	–	–	–	–
Gallstone	5.3 (1.8–12.9)	0.002	–	–	–	–
Choledochal cyst	29.7 (2.3–NE)	0.041	29.7 (2.3–NE)	0.041	29.7 (1.8–489.5)	0.048
Liver mass/metastasis	35.8 (0.8–NE)	0.20	–	–	35.8 (0.8–1600)	0.073
Bladder wall thickening	104.9 (0.5–NE)	0.25	–	–	–	–
Bladder polypoid lesion	7.8 (0.9–67.5)	0.39	12.4 (0.7–220.1)	0.093	10.9 (0.6–192.5)	0.11
Adrenal lesion (>4 cm)	15.8 (0.8–NE)	0.24	15.8 (0.8–312.5)	0.074	13.6 (0.7–271.4)	0.089
Pancreatic lesion	110.0 (0.6–NE)	0.36	–	–	–	–
Large ovarian/uterine mass	17.0 (5.3–54.5)	<0.001	12.9 (3.0–55.2)	0.001	11.4 (2.5–51.4)	0.002
Ovarian solid lesion	NE (0.0–NE)	0.27	–	–	–	–
Lymphoma (suspected)	110.5 (0.8–NE)	0.051	–	–	–	–

Odds ratios (OR) and 95% confidence intervals (CI) are shown. NE (Not estimable) indicates that OR and/or CI could not be calculated because no events occurred in the comparison group; *p*-values in these cases were derived from Fisher’s exact test. The multivariate model included only variables of clinical relevance and/or those with *p* < 0.10 in univariate analysis, considering the limited number of cancellation events (n = 11). Very high univariate OR values for common findings (hepatomegaly, steatosis, gallstones) did not persist as independent predictors after adjustment, reflecting the effect of small sample size and collinearity. Firth’s penalized logistic regression was applied to reduce small-sample bias in rare-event estimation.; USG, ultrasonography; OR, odds ratio; CI, confidence interval; NE, not estimable.

## Data Availability

The datasets generated and/or analyzed during the current study are available from the corresponding author upon reasonable request.
